# Time-Reversible Implementation of MASH for Efficient Nonadiabatic Molecular Dynamics

**DOI:** 10.1021/acs.jctc.4c01684

**Published:** 2025-02-25

**Authors:** J. Amira Geuther, Kasra Asnaashari, Jeremy O. Richardson

**Affiliations:** Department of Chemistry and Applied Biosciences, ETH Zurich, Zurich 8093, Switzerland

## Abstract

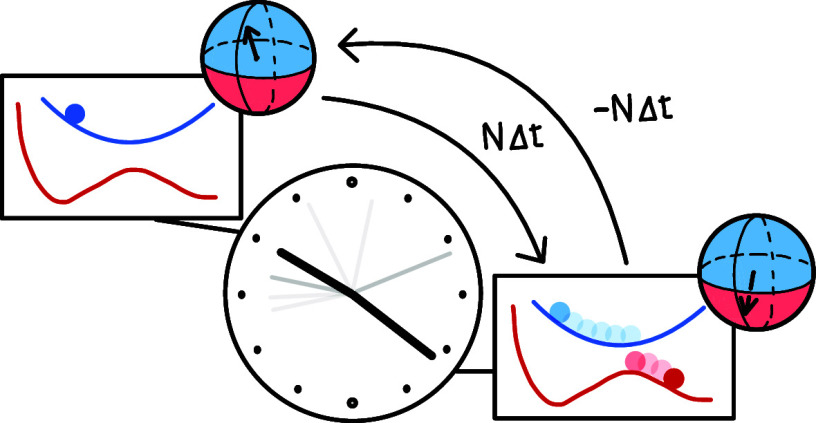

In this work, we describe various improved implementations of the mapping approach to surface hopping (MASH) for simulating nonadiabatic dynamics. These include time-reversible and piecewise-continuous integrators, which are only formally possible because of the deterministic nature of the underlying MASH equations of motion. The new algorithms allow for the use of either wave-function overlaps or nonadiabatic coupling vectors to propagate the spin, which encodes the electronic state. For a given time-step, Δ*t*, it is demonstrated that the global error for these methods is  compared to the  error of standard implementations. This allows larger time-steps to be used for a desired error tolerance, or conversely, more accurate observables given a fixed value of Δ*t*. The newly developed integrators thus provide further advantages for the MASH method, demonstrating that it can be implemented more efficiently than other surface-hopping approaches, which cannot construct time-reversible integrators due to their stochastic nature.

## 1 Introduction

Molecular dynamics (MD) simulates the behavior of atoms and molecules, providing insights into the behavior of matter at the atomic level, which is crucial for understanding chemical reactions, material properties, and biological processes.^1^ A fundamental concept of standard MD is the Born– Oppenheimer approximation,^2^ which assumes that the electronic and nuclear motion can be separated by evolving the electronic wave function adiabatically along the nuclear trajectory. However, this approximation breaks down in situations where the potential energy surfaces of different electronic states come close to each other or intersect, leading to strong coupling between electronic and nuclear degrees of freedom. These scenarios, known as nonadiabatic processes, are essential in understanding and describing photochemical reactions, electron-transfer processes, and the behavior of excited states in materials.^3^

Unfortunately, solving the full time-dependent Schrödinger equation is not possible for most systems of interest as the computational cost for fully quantum-mechanical methods increases exponentially with the size and complexity of the system studied. Thus, several mixed quantum−classical methods have been developed to describe nonadiabatic processes based on independent trajectories, such as various mapping approaches^4,5^ including spin mapping.^6,7^ However, the most commonly used approach in molecular photochemistry is fewest-switches surface hopping (FSSH).^8^ This method propagates the classical nuclei on a single adiabatic energy surface while stochastic hops allow for a switch between different electronic states. It correctly describes certain effects in nonadiabatic processes such as wave-packet branching^9^ although it also has several known problems such as an inconsistency with its wave-function coefficients.^8,10^

Recently, we have developed the mapping approach to surface hopping (MASH).^11,12^ It combines concepts from both spin mapping and surface hopping but shows distinct advantages over those methods in terms of rigor and accuracy at a comparable computational cost. In particular, unlike FSSH,^13,14^ MASH captures the correct rate in the Marcus-theory limit without requiring complicated decoherence corrections,^15^ and it appears to outperform all forms of FSSH^a^ and even AIMS (ab initio multiple spawning)^17^ in first-principles simulations of photochemistry.^18^ One argument for why MASH shows this good behavior is because it can be rigorously derived from a short-time limit of the quantum– classical Liouville equation (QCLE), as we have discussed in previous work.^11,12^ Similarly to other surface-hopping methods, MASH is therefore valid as long as long-time electronic coherence and nuclear quantum effects can be neglected, as is commonly the case for typical applications in molecular photochemistry.

This work does not focus on the theoretical accuracy of MASH but rather on the numerical accuracy of its implementation. In particular, we construct time-reversible integrators, explore different methods to compute the time-derivative couplings and implement variable time-stepping. Note that, this is only possible due to the deterministic nature of the theory, which yields reversible equations of motions that allow one to “undo” propagation steps as well as to reverse the dynamics. Such properties do not hold for FSSH, as this does not have time-reversible equations of motion, and it is also not easy to “undo” a propagation step once one has drawn a random number without biasing the stochastic process.

We will begin with a brief explanation of the MASH method. Following this, we describe various ways to propagate the spin vector. These are combined with standard methods for nuclear propagation to construct time-reversible and piecewise-continuous algorithms. Finally, we present results from tests on model systems to compare the efficiency of the new algorithms to the previously used nonreversible MASH algorithms, which were based on standard approaches used for FSSH.

## 2 Mapping Approach to Surface Hopping

As the original MASH approach is only applicable to two-level systems, we focus on this case. However, our integrators can easily be generalized to treat the multistate MASH methods so far proposed.^19,20^

In many ways, MASH is similar to FSSH: the nuclei are evolved on a single active adiabatic potential energy surface and can switch surface by jumping to a different state. While the nuclei are treated classically with coordinates ***q*** and associated momentum ***p***, the electronic state is treated quantum mechanically, either using wave-function coefficients, or equivalently by a spin vector ***S*** = (*S*_*x*_, *S*_*y*_, *S*_*z*_) on the Bloch sphere. In MASH, rather than using a stochastic process, the active state is chosen deterministically according to the instantaneous position of the spin vector. If the spin is in the southern hemisphere of the Bloch sphere, the nuclei should be evolved on the lower adiabatic energy surface, *V*_0_(***q***); if it is in the northern hemisphere, they are propagated on the upper surface, *V*_1_(***q***). The two states interact via the nonadiabatic coupling vector (NAC), ***d***(***q***), defined by the elements
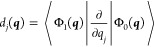
1 where Φ_0_ and Φ_1_ are the stationary, real-valued electronic wave functions of the two adiabatic states.

The equations of motion are (with )^11^
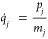
2a
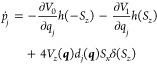
2b
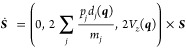
2c where *m*_*j*_ is the mass associated with the *j*th degree of freedom and . The Heaviside step function, *h*(*S*_*z*_), determines the active surface and hence the force felt by the nuclei. When *S*_*z*_ passes through zero (the Bloch sphere’s equator), the electrons attempt to hop to the other state and the nuclei feel an impulse in the direction of the nonadiabatic coupling vector ***d***(***q***) due to the Dirac delta function, . This ensures that the energy
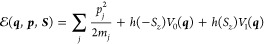
3is conserved. If not enough energy is available to hop up, the attempt is rejected and the momenta are reflected along the direction of the NAC, which was also the suggestion first made by Tully as noted in ref (21).

Note that MASH is defined in the kinematic representation (where one uses adiabatic states with the kinematic rather than the canonical momentum)^22^ and its dynamics are thus not generated by a Hamiltonian. One cannot therefore hope to obtain a symplectic integrator (at least not with a canonical structure), as has been achieved for mapping and spin-mapping methods.^23,24^ Unlike other surface-hopping methods,^25^ however, the MASH equations of motion are fully deterministic and time-reversible (as is clear from the replacement ). The fact that the differential equations are time-reversible inspires us to search for a numerical implementation which retains this symmetry with arbitrary step sizes.

### 2.1 Nuclear Propagation

Before tackling the coupled case, we first consider propagating the nuclei and spin vector separately. In particular, it is clear that the nuclei can be evolved using the standard velocity-Verlet algorithm^26^ given by
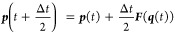
4a
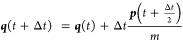
4b
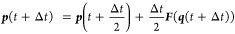
4cwhere ***F*** is given by
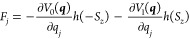
5

There are a number of ways of propagating ***S***, which we discuss later. In any case, after each update, we test for hops by comparing sgn(*S*_*z*_) before and after the step. After a hop, the momentum is rescaled. The rigorous connection between MASH and the QCLE gives a unique procedure for rescaling the mass-weighted momentum () along the direction of the mass-weighted NAC (). The total available energy is given by
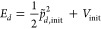
6where  and *V*_init_ are the initial momentum along the NAC and the initial potential before the hop. To determine whether there is enough energy available to accept the hop, the final potential energy, *V*_fin_, is computed on the new surface. If , the hop is accepted and the momentum is rescaled according to
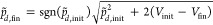
7to give
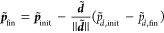
8

If , the hop is rejected and the nuclei are reflected by inverting the component of the momentum along the nonadiabatic coupling, giving
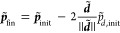
9

Finally, each degree of freedom, *j*, is multiplied by  to give the full, rescaled momentum . Additionally, the *S*_*z*_ coordinate is inverted so as to remain in the initial hemisphere.

### 2.2 Spin Propagation with NACs

The simplest method to propagate the spin vector according to its equation of motion Eq 2c is
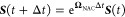
10with
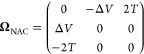
11where
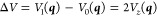
12and
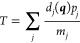
13

This approach is particularly simple to implement (assuming NACs are available) as it only requires information from one electronic-structure calculation at a time. However, the well-known disadvantage of using NACs is that they can be strongly peaked for systems with weak diabatic couplings.^27,28^ This means that a very short time-step may be necessary to simulate such systems correctly.

### 2.3 Spin Propagation with Averaged Time-Derivative Couplings

An alternative to directly using NACs is to reformulate the theory in terms of wave-function overlaps. The advantage of this is that it will correctly capture the effect of narrow couplings which were passed between *t* and *t* + Δ*t* without requiring a short time-step.^27^ It does, however, bring an extra complication that knowledge of the wave functions at two points in time are required, which, as we will see later, makes constructing reversible integrators more difficult.

The overlap matrix is defined as
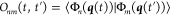
14

Following Jain et al.,^29^ we note that
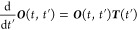
15 where the time-derivative coupling matrix has elements
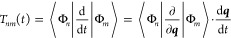
16

In the two-state case these can be written as^b^
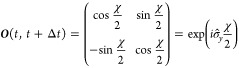
17where  is a Pauli matrix and
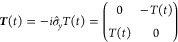
18where *T*(*t*) was defined in Eq 13. It is necessary to impose a sign convention such that the wave functions change smoothly along the trajectory. We choose the arbitrary global phase (or the sign in our real-valued case) of each wave function such that it has a positive overlap with the corresponding state at the previous step. This ensures that the diagonal elements of the overlap matrix are positive and therefore .

The solution of Eq 15 is
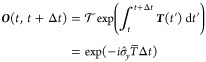
19 where the time-ordering operator, , is not required in the two-state case as  commutes with itself at different times. We are thus naturally led to defining the averaged time-derivative coupling as
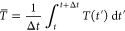
20

Putting all this together, we obtain
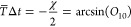
21which is the formula used in practical applications to obtain the averaged time-derivative coupling directly from the overlap matrix.

To propagate the spin vector, we write
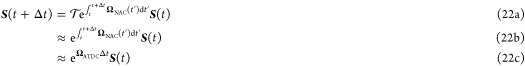
where in the second line we have approximated the time-ordered exponential by an ordinary exponential, which is valid only for small Δ*t*. The final line defines the method used in practice, where
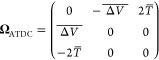
23and the integral over Δ*V* implied by Eq 22b is approximated by the trapezium rule:
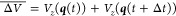
24

This is the method which was used in our previous ab initio MASH simulation of cyclobutanone.^30^ It is strongly based on the approach of Jain et al.,^29^ who wrote the averaged time-derivative coupling in terms of a logarithm of the overlap matrix. In turn, their approach was based on the original idea of Meek and Levine’s norm-preserving interpolation.^27^ The concept behind this was effectively to approximate *T*(*t*′) by a linear function along the time-step. Although this has the advantage of capturing the overall effect of a narrow avoided crossing, we see no physical reason for assuming *T*(*t*′) to be a linear function, especially in the vicinity of a narrow avoided crossing where it is strongly peaked. We have therefore chosen not to name our method the “norm-preserving interpolation,” as all the methods presented in this paper correctly preserve the norm of the spin vector, and additionally we have simply averaged the time-derivative coupling, rather than interpolating it. We will thus refer to this method as the averaged time-derivative coupling (ATDC) approach.

A similar derivation starting from a Trotter splitting of Eq 22a gives
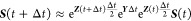
25where
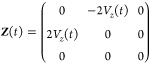
26a
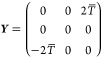
26b

We will not use this approach directly in this work, as it turns out to be identical to the local-diabatization method described next.

### 2.4 Spin Propagation Using Local Diabatization

Plasser, Granucci et al.^28^ offer a third alternative, known as the local-diabatization (LD) approach. Here, one treats the adiabatic states as if they were the basis of a (diagonal) diabatic representation using ***Z***(*t*) as defined in Eq 26a. However, to recover the correct propagation, one must take account of the fact that the basis at time *t* is different from those at time *t* + Δ*t*. Therefore, the spin is updated by taking half of a time-step in the initial basis, rotating to the new basis and finally taking half a time-step in this basis:
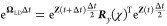
27

The rotation matrix
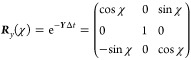
28is written in terms of the rotation angle χ as defined in Eq 17.

This is almost a faithful reformulation of the method of Plasser, Granucci et al.^28^ from the language of wave functions into that of spin vectors. There is however one small modification that we have introduced, to use
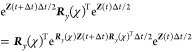
 rather than
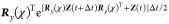
which would correspond exactly to the original suggestion. Our approach is expected to be a slightly more accurate approximation to the time-ordered exponential of Eq 22a. Even more accurate versions can be constructed by taking multiple small steps using propagators obtained by interpolating between ***Z***(*t*) and ***R***_*y*_(χ)***Z***(*t* + Δ*t*)***R***_*y*_(χ)^T^. In fact, an analytic solution could probably be found in terms of Weber functions, following Zener’s famous derivation of an equivalent problem.^31^ Although these improvements may achieve a small gain in accuracy, they do not in general increase the order of the method, as the assumption of linear interpolation makes the method second order for any nonlinear Hamiltonian.

We find that the local-diabatization approach is more accurate than the approaches based on Meek and Levine’s idea of linearly interpolating the time-derivative coupling.^27,29^ Only the local-diabatization method can be made exact for the simple case of a Landau–Zener model, as the interpolation is perfect for a linear diabatic Hamiltonian. The local-diabatization method is also the preferred approach in other surface-hopping implementations.^16^ However, as we have shown there is no fundamental difference between the local-diabatization and the ADTC approaches, as the latter can be made equivalent to the former by simply starting from a Trotter splitting of the propagator as in Eq 25.

A simple alternative to these approaches is available for model systems defined in terms of a global diabatic representation. This is exploited in Runeson’s simulations of exciton dynamics.^19^ We will, however, focus on the standard situation for ab initio simulations of photochemistry, in which a global diabatic representation is not available.

Finally, we note that we see no need to employ multiple-time-stepping algorithms to propagate the electrons with a smaller time-step than the nuclei. We suppose that one reason that such approaches were previously favored is due to the use of Runge–Kutta solvers, rather than propagators based on matrix exponentials.^21,29^ However, there is perhaps also a deeper reason, that in FSSH, the probability of hopping during an individual step is based on a short-time approximation which depends on the rate of change of the wave-function coefficients.^8^ It is therefore necessary to employ small time-steps to ensure that the algorithm attempts hops during the period when the coefficients are changing. In MASH, an attempted hop can be detected simply from a switch of the sign of *S*_*z*_ and can therefore take larger steps without causing problems.

## 3 Implementation

Having discussed the various possible ways to update the nuclei and the spin vector separately, we now consider how to put these together to obtain efficient integrators for the full MASH dynamics. Although the equations of motion for MASH are formally time-reversible, current ab initio implementations of MASH^18,20,30^ are based on algorithms originally developed for surface hopping, which are not time-reversible.^16^ For instance,


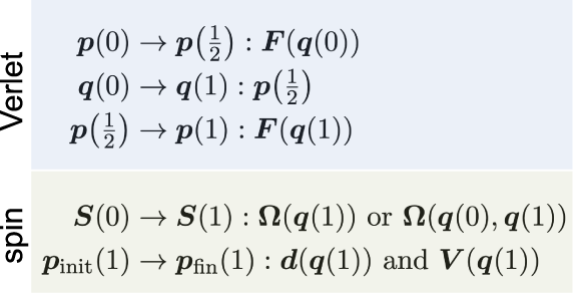
where 0 and 1 denote times *t* and *t* + Δ*t* and the rest of the notation is self-explanatory. For instance, in the first line ***p*** is propagated forward by half a time-step according to the force measured at ***q*** at the start of the step.

This nonreversible approach can be applied using either NACs or overlaps to propagate the spin vector. When propagating with NACs, there are two ways to define , either based on information at ***q***(1) only (which we call asym-NACs), or the more accurate approach of averaging the information at ***q***(0) with ***q***(1), i.e.,  (which we call nonrev-NACs). For the overlap methods called nonrev-ATDC and nonrev-LD, the **Ω** matrix is necessarily defined by both points. In all cases, the momenta are rescaled after attempted hops in the final line, which requires the calculation of the adiabatic energies and NAC at ***q***(1). All these algorithms correctly reproduce MASH dynamics in the limit of Δ*t* → 0 and some have been used in ab initio simulations.^30^

Nonetheless, it is desirable to develop a time-reversible algorithm, as it is nearly always a good thing to preserve a symmetry wherever possible. In particular, in the long-time limit, it is expected that time-reversible integrators give more reliable results using larger time-steps.^26^ The trick for obtaining a time-reversible integrator is to construct it in a symmetric way such that it reads the same forward as backward. A simple example for this is the velocity-Verlet algorithm which updates momenta, positions and then momenta again. This concept can be extended to obtain symmetric integrators for MASH, in which the phase space additionally includes the spin vector.

### 3.1 Reversible NACs Approach

To build a time-reversible algorithm using the nonadiabatic coupling vectors, the propagation of the spin ***S*** can be divided into two half-steps that are built around the velocity-Verlet algorithm. This is possible because knowledge of the wave functions at only one point in time is necessary to propagate ***S*** when using NACs. After each update of ***S***, we check whether a hop has occurred, in which case the momentum is rescaled or inverted.


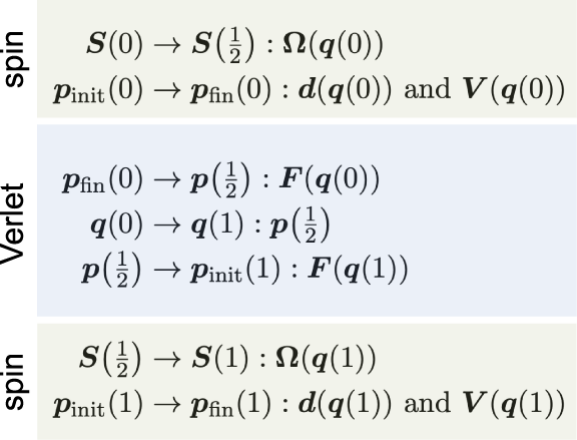
Unlike the asymmetric algorithm presented above, it is clear that if you run a trajectory backward in time using this reversible algorithm (either by using a negative value of Δ*t* or by flipping the signs of ***p*** and *S*_*y*_), it will retrace its steps perfectly. This is true (apart from round-off error) even for large values of Δ*t* and is confirmed by numerical tests.

### 3.2 Reversible Piecewise-Continuous Approaches

The reversible NACs algorithm cannot be directly generalized to use overlaps. This is because constructing the overlap requires knowledge of the wave functions at two points in time. It is therefore not possible to update the spin before the nuclei. It is thus necessary completely to restructure the algorithm when using overlaps.

To proceed, we first notice that the standard algorithms (nonrev-NACs, nonrev-ATDC, nonrev-LD) are actually time-reversible for any step in which no hops occur. This is because during these steps, the nuclear variables and the spin vector are not coupled and the propagators for these two separate parts are themselves reversible. In particular, the nuclei are propagated using the time-reversible velocity-Verlet algorithm. In order to prove that the spin update is reversible, we need to demonstrate that the propagator which takes ***S***(*t*) to ***S***(*t* + Δ*t*) is equal to its adjoint.^26^ The adjoint of a propagator  is defined as . In other words, propagating backward in time should be the inverse of forward propagation. This is easy to show for nonrev-NACs, where **Ω**_NAC_ is defined as the average of the information from the initial and final points. Similarly, nonrev-ATDC makes use of time-averages, Eq 24 and Eq 21, which are symmetric to time reversal, i.e., **Ω**(*t*, *t* + Δ*t*) = **Ω**(*t* + Δ*t*, *t*) such that e^**Ω**(^^*t*^^,^^*t*^^+Δ^^*t*^^)Δ^^*t*^ = (e^–**Ω**(^^*t*^^+Δ^^*t*^^,^^*t*^^)Δ^^*t*^)^–1^. Finally, it can be shown that the propagator of nonrev-LD, Eq 27, obeys:
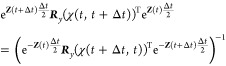
This follows because (***R***_*y*_(*χ*(*t* + Δ*t*, *t*))^T^)^−1^ = ***R***_*y*_(*χ*(*t*, *t* + Δ*t*))^T^ due to the properties of orthogonal rotation matrices and because *χ*(*t*, *t* + Δ*t*) = –*χ*(*t* + Δ*t*, *t*) due to the antisymmetry of the overlap matrix. Taken together, these arguments demonstrate that the spin is evolved in a time-reversible fashion by all these methods, i.e., propagating forward and then backward will return the spin exactly to its initial value.

When the sign of *S*_*z*_ changes within a time-step, however, this is only noticed by the nonreversible algorithm at the end of the step. As a result, the nuclei are propagated on a single electronic surface for the full step. When this step is reversed, the nuclei again evolve on a single surface, but this time on the other surface, causing a mismatch between forward and backward trajectories.

We will therefore use the standard algorithm as our building block and adjust it in a way to ensure time-reversibility even in steps that include hops. This is achieved by dividing the step into two parts at the time of hopping τ. The nuclei and spin are first propagated until τ, using the standard algorithm. Then, the hop is performed and the momentum is rescaled before the standard algorithm is applied again to propagate to the end of the step:


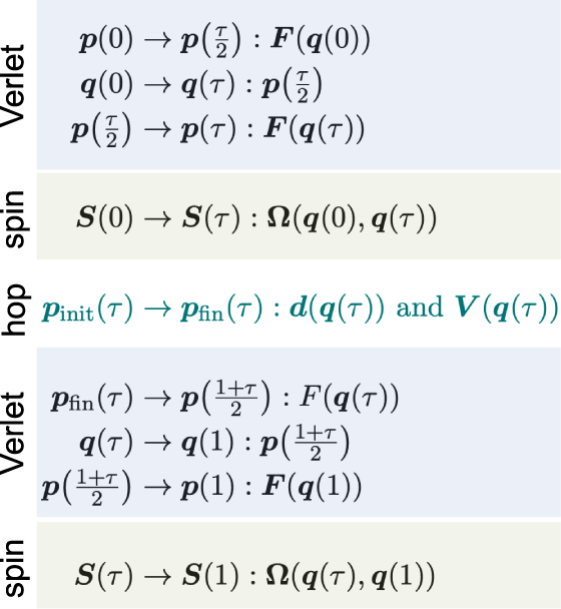
Note that only one rescaling step is needed as we assume that maximally one hop can occur during a full time-step. Numerical tests show perfect agreement between the forward and backward propagation, confirming that the algorithm is time-reversible. We call these methods piecewise-continuous (pc) as the discontinuity introduced by the hop is carefully controlled between two continuous evolutions. Various spin-propagation approaches can be used, leading to the methods rev-pc-NACs, rev-pc-ATDC and rev-pc-LD. To ensure that rev-pc-NACs is reversible, it is necessary to average the NACs between 0 and τ or τ and 1 as appropriate, similarly to nonrev-NACs.

**Figure 1:**
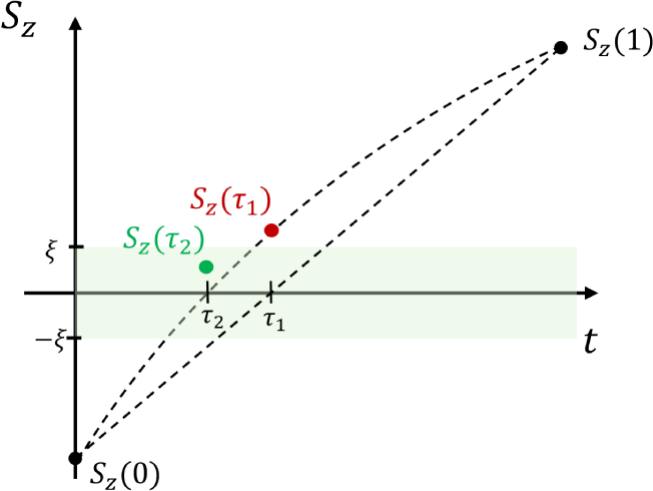
Example of a root search for the hopping time, τ; after a state change has been detected, the first estimate is τ_1_, but *S*_*z*_(τ_1_) does not lie within the error band. Thus, a new estimate τ_2_ is made using a spline interpolation between *S*_*z*_(0), *S*_*z*_(τ_1_) and *S*_*z*_(1). This fulfills the condition given by ξ and the search is finished.

These methods of course require that one knows the time, τ, at which the hop will occur. This point can be found using any one-dimensional root search. In this work, we employed the following algorithm: First, the propagation is performed for the full time-step and the state before and after the step are compared to check for hops. If there was no change, the step is finished. If there is an attempted hop, an estimate of τ (called τ_1_) is made using a linear interpolation between *S*_*z*_(0) and *S*_*z*_(1). The propagation is then recalculated from *t* = 0 to *t* = τ_1_ using the steps as shown above. If *S*_*z*_(τ_1_) now lies within an error band around the equator of the Bloch sphere specified by the parameter ξ, ***p*** is rescaled and the variables are propagated to *t* = 1 to finish the step. If *S*_*z*_(τ_1_) does not fall into this region, a better estimate for τ (called τ_2_) is calculated using spline interpolation (up to third order) between all values of *S*_*z*_ that are obtained so far. This procedure is iterated until convergence (see Figure 1). It is important to emphasize that there are only two steps in the final propagation, divided at the converged value of τ.

Overall, the algorithm becomes rigorously time-reversible in the limit that ξ → 0. We use a value of ξ = 10^–4^ and find that this typically requires only about 2–3 iterations to obtain a sufficiently converged value of τ. Numerical tests confirm that trajectories retrace their steps almost perfectly when time is run backward (as demonstrated in Section 4). The piecewise-continuous methods are formally implicit, meaning that an iterative approach is required. Implicit approaches are not commonly favored in molecular science as they increase the overall cost of the simulation.^26^ However, in our case, the iteration is only required on steps which hop and as hopping events are rare on the time scale of a full trajectory, the increase in computational cost from these iterations is not noticeable. As we shall show, the benefits far outweigh the extra complexities.

## 4 Results and Discussion

This paper has introduced new time-reversible methods, based either on nonadiabatic coupling vectors (rev-NACs and rev-pc-NACs) or wave-function overlaps (rev-pc-ATDC and rev-pc-LD). We shall compare these with the original nonreversible algorithms (asym-NACs, nonrev-NACs, nonrev-ATDC, nonrev-LD) to illustrate the differences in accuracy for various choices of time-step, Δ*t*.

To illustrate the reversibility of the new methods we analyzed individual trajectories run forward and then backward in time. Moreover, we implemented variable time-stepping which is only possible due to the deterministic nature of the theory. This approach employs a large time-step for the simulation which is divided into smaller steps whenever the large time-step results in inaccuracies. This requires that a condition is checked after each step to determine whether significant errors were made. For instance, as the velocity-Verlet algorithm itself is not exactly energy conserving, the conservation of energy can be a useful indicator for inaccurate propagation due to a too large time-step.

**Figure 2:**
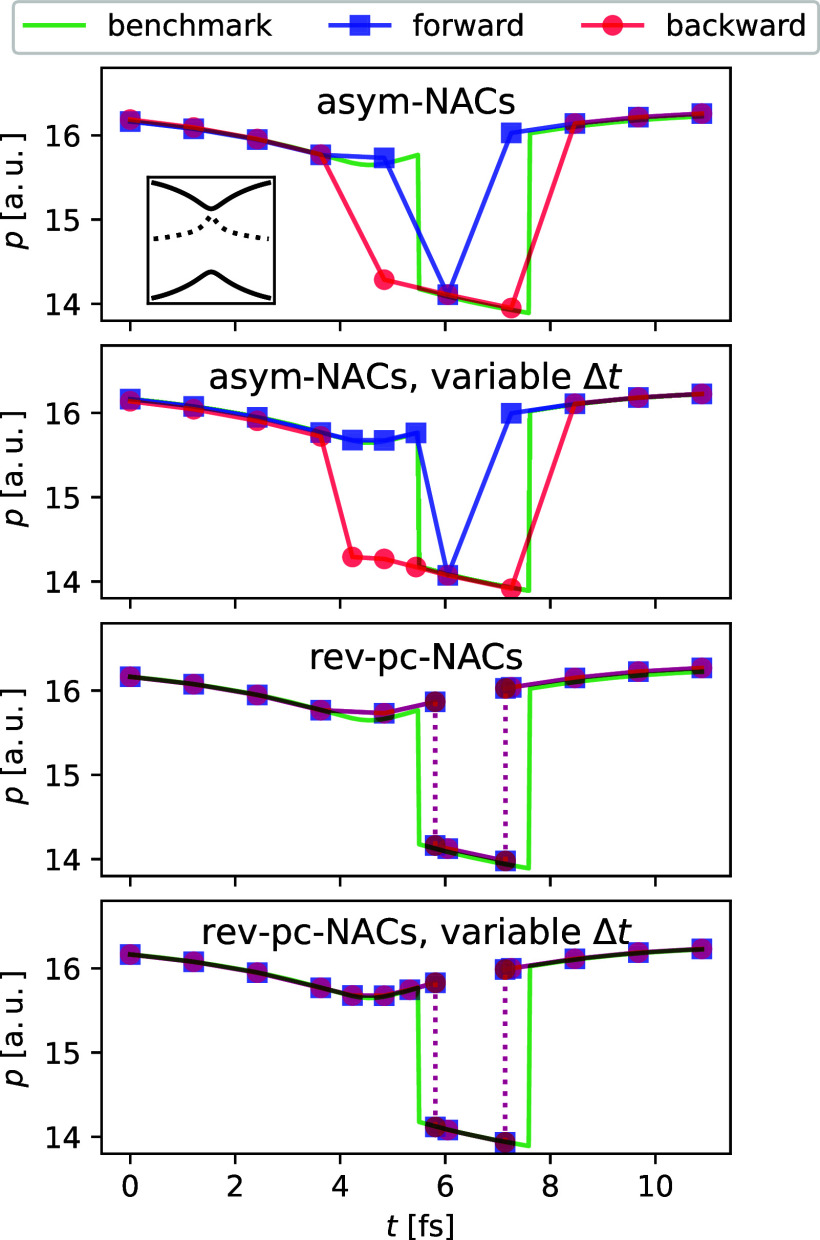
Example trajectory for the propagation forward and backward in time on the Tully model with initial conditions *q*(0) = −1.5, *p*(0) = 16.16, and the normalized vector of ***S***(0) = [0.02, 0.056, −0.998] . The potential is shown in an inset with the PES in solid and the NAC in dotted lines, scaled by a factor of 1/400. Each method is compared to a benchmark trajectory computed at a smaller time-step where all methods agree. The variable time-stepping algorithm recursively bisects steps in which . In the plot, the size of the time-steps taken is indicated by the spacing of the dots.

In Figure 2, we show an example trajectory propagated first forward and then backward in time on Tully’s simple model of an avoided crossing^8^ using asym-NACs and rev-pc-NACs, with and without variable time-stepping. One can see that the rev-pc-NACs method retraces its steps almost perfectly (better than 1 part in a million), while the asym-NACs method shows a large difference between the forward and backward trajectories, which is not completely resolved even after both trajectories hop twice. The situation is even worse in other cases where the number of hops differs (not shown). Using variable time-stepping yields only minimal improvements but does not resolve the discrepancy between forward and backward asym-NACs trajectories and does not correct the small error in the hopping time of rev-pc-NACs. Overall, these results indicate that it is not particularly helpful to use variable time-stepping in this case. It is, however, clear that more accurate simulations are obtained using reversible integrators than variable time-stepping with the standard approaches.

### 4.1 Error Analysis

When we discuss the error made by an integrator, we must distinguish between the truncation (local) error of a single step and the global error at a specified time after many steps. The concatenation of local errors typically causes the global error to appear at a lower order of Δ*t*. For example, the velocity-Verlet algorithm has  truncation error. However, it is known as a second-order method as its global error is .^26^ This is justified by a simple argument. Using  to represent the propagator (known as a dynamical map): , where *N* = *t*_max_/Δ*t* is the number of time-steps used to propagate to time *t*_max_. Expanding using the binomial series gives 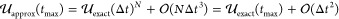
as we wished to show.

The simple reason for the excellent behavior of the velocity-Verlet algorithm (relative to alternative approaches such as the Euler method) is due to its time-reversible nature. This symmetry ensures that the order of the method is even. In contrast, nonreversible algorithms may have odd order. Therefore, unless one works hard to construct a complicated high-order algorithm, the global error will typically be , i.e., a first-order method.

A formal error analysis could of course be carried out mathematically for our proposed algorithms. However, this is complicated by the fact that MASH trajectories are not continuous, let alone twice differentiable as is required by textbook derivations.^26^ It is therefore much simpler to present illustrative examples.

We again employed the Tully model of an avoided crossing and read off the order of error from the numerical data. We consider results from both single-trajectory calculations as well as averages over many trajectories. In the latter case, the initial spin vector ***S*** was sampled uniformly from the lower half of the Bloch sphere (with unit radius) and the nuclear coordinates were sampled from the Wigner distribution
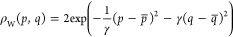
29

The order of the global error can be obtained numerically from the slope of a logarithmic plot of the error vs Δ*t*. In this work, we define the global error for a trajectory as
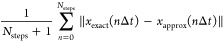
where *x* can be any of the phase-space variables (position, momentum, or spin) and *x*_exact_ is a benchmark trajectory run at a very small time-step where all of the methods agree.

**Figure 3:**
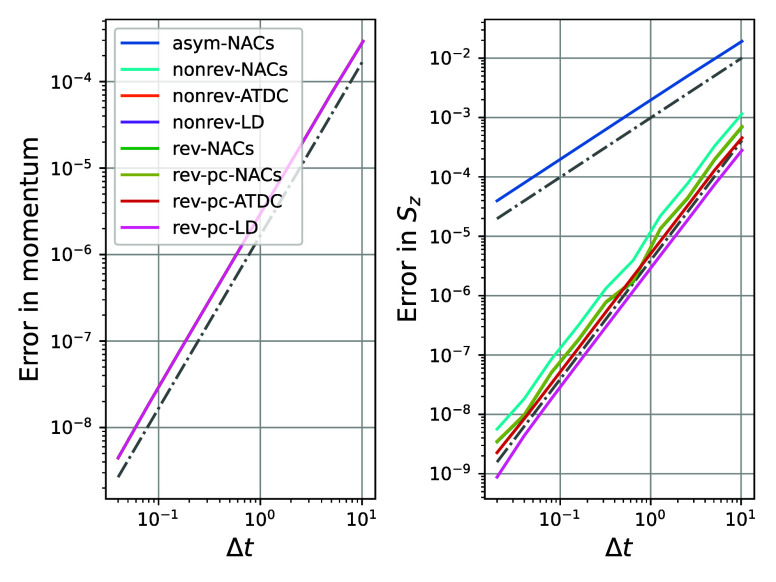
Comparison of the order of the global error for the different implementations of MASH for a generic trajectory without hops going from left to right (*q*_0_ = −1.5, *p*_0_ = 15.6, ***S*** = [−0.04, −0.08, 0.9]) in the Tully model. All methods show a global error of order two for the momentum due to the underlying velocity-Verlet algorithm (all colored lines are on top of each other in the left graph). However, only the symmetric methods are of order two for *S*_*z*_. The dot-dashed gray lines have slopes of 1 and 2 for comparison. Abbreviations are defined as follows: algorithms based on NACs comprise asymmetric (asym, which use information only at one time point), nonreversible (nonrev), reversible (rev) and reversible piecewise-continuous (rev-pc) forms; nonreversible and reversible piecewise-continuous algorithms based on overlaps may either use the averaged time-dependent coupling (ATDC) or the local diabatization (LD) approach.

We first look at the case of a trajectory in which no hops occur. It is important to notice that in this case, position and momentum are updated exclusively by the velocity-Verlet algorithm, which is second order. This is illustrated clearly by the second-order dependence of the error in the momentum on the time-step as shown in Figure 3 a. However, the methods differ in the way they update the spin vector. In particular, when one looks at the *S*_*z*_ variable, shown in Figure 3 b, it is clear that the asym-NACs algorithm is only first order, whereas all the other (symmetric) algorithms are second order. Note that in this case it is not the NACs method *per se* that causes the poor performance, but rather the asymmetry of the spin-propagation algorithm. This example shows clearly how much higher accuracy can be achieved using symmetric algorithms for the same choice of time-step.

Additionally, we note that in this case the LD method is slightly more accurate than the ATDC or NACs approaches. In cases where the avoided crossing is even sharper (not shown), the NACs method performs significantly worse than the two methods based on overlaps, as expected from previous work.^27,28^

**Figure 4:**
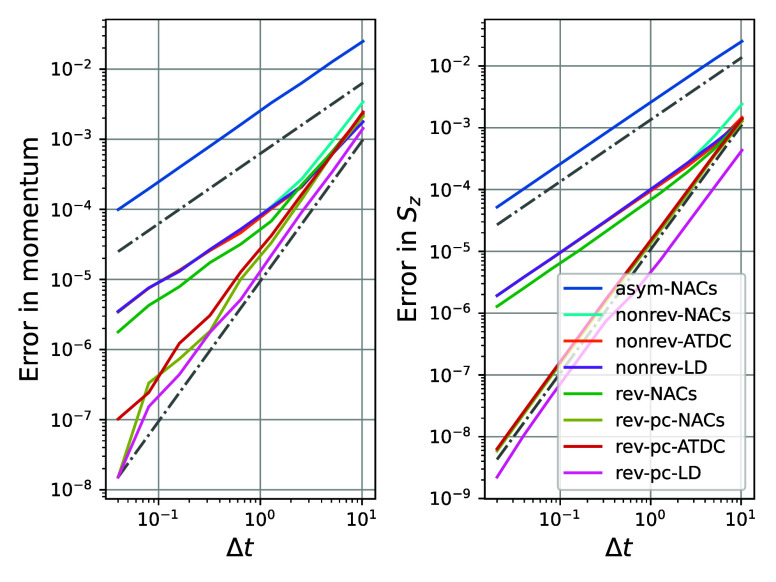
Comparison of the order of the global error for the different implementations of MASH averaged over 5000 trajectories sampled from a Wigner distribution centered around  and  with γ = 0.1. Some trajectories do not hop, others hop once, twice or more, with an average of about one hop. Only the piecewise-continuous methods preserve the second-order accuracy while the rev-NACs and nonreversible methods become first order due to the  error made during hops.

The same process was repeated for an ensemble of trajectories which included hops. To ensure a fair comparison, exactly the same set of initial conditions was used for each method. The results are shown in Figure 4.

The first thing to notice is that due to the coupling between nuclear and electronic degrees of freedom, even the error in momentum becomes first order for the nonrev-NACs method. We also see that other nonreversible methods (nonrev-ATDC and nonrev-LD) are first order. It is perhaps surprising at first sight that the rev-NACs method also appears to be first order, even though it is a time-reversible method. This observation is explained by noting that the textbook global-error analysis does not directly apply to MASH algorithms as the momenta are not continuous across a hop. Each time a hop is encountered by these methods, an error of  is made. Assuming there are only a finite number of hops along a trajectory implies that the global error will also be of , as confirmed in the results.

The only methods which remain second order are the reversible piecewise-continuous algorithms. These methods avoid the difficulty of the discontinuity by dividing the propagation into two continuous steps, as the name suggests. It does not introduce an  error when hops are performed and thus preserves the second-order nature of the methods. This results in an increased accuracy of several orders of magnitude.

### 4.2 Example Application to Pyrazine

After formally analyzing the order of the error, we next test the implementations on a more relevant chemical problem in multiple dimensions. For this, we chose to simulate the internal conversion in a 2-state 3-mode linear-vibronic-coupling model of pyrazine.^32^ We initialized the simulations from a harmonic Wigner function in the higher diabatic state and measured the decay of this diabatic population. Diabatic observables are obtained according to the standard MASH procedure as linear combinations of correlation functions of adiabatic populations and coherences including the appropriate weighting factors.^11,12^ A comparison with exact quantum-mechanical results is presented in previous work, showing that MASH gives a reasonable approximation.^11^ Here, we focus only on the numerical accuracy of the implementation.

**Figure 5:**
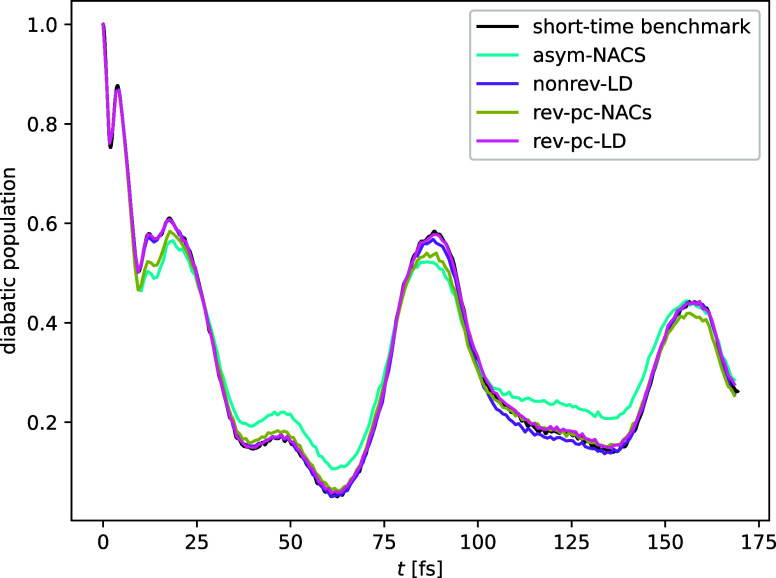
Comparison of the time-dependent diabatic population for a 2-state 3-mode model of pyrazine computed with Δ*t* = 1.2 fs to a benchmark obtained with Δ*t*_0_ = Δ*t*/350, where all methods agreed. The reversible, piecewise-continuous methods outperform their nonreversible counterparts. Additionally, the LD methods based on overlaps perform significantly better than those based on NACs. ATDC (not shown) is similar to LD in this case.

To test the accuracy of the various algorithms, a benchmark result was obtained as the average of 100,000 trajectories with a very small time-step, Δ*t*_0_, where agreement was seen between all methods. The calculation of the trajectories was then repeated with the same set of initial conditions for a large time-step Δ*t*. As shown in Figure 5 the reversible, piecewise-continuous version of each algorithm is more accurate than its nonreversible counterpart. Moreover, the rev-pc-LD algorithm performs best and shows almost perfect agreement with the benchmark, whereas the nonrev-LD deviates in the regions of 80–90 and 100–140 fs. Both NACs methods (and especially asym-NACs) perform significantly worse, which is expected as the well-known disadvantage of using NACs is that they can be strongly peaked for systems with weak diabatic couplings such as in the vicinity of a conical intersection.^27^ This means that a short time-step may be necessary to reproduce the correct dynamics. Overall, these results show that the new reversible implementations of MASH allow for a larger time-step with no loss in accuracy.

Moreover, we also employed variable time-stepping but found that it does not noticeably improve the accuracy of the simulations. Instead, even with a low threshold for the maximum allowable change in the energy, the performance showed no noticeable improvement, despite incurring three times the computational cost. This implies that one should look for a better indicator than energy conservation to detect when smaller time-steps are required. For example, one could use the smaller time-steps only when hops are encountered. However, we have shown that variable time-stepping is not necessary when using the new reversible, piecewise-continuous methods. Additionally, locating the exact time of hopping is more efficient using interpolation than bisection^19^ traditionally used in the variable time-stepping approach.

## 5 Conclusion

This work has enhanced the mapping approach to surface hopping (MASH) by developing time-reversible, second-order integrators. This improves the accuracy of the trajectories for a given finite step-size, allowing larger time-steps to be made for a given error tolerance.

Important to note is that constructing a time-reversible integrator alone is not sufficient to ensure second-order accuracy. This subtle point arises because of the discontinuity of MASH trajectories when the electronic state is changed, which introduces an error of  whenever a hop occurs. This lowers the order of the global error of rev-NACs to first order. In order to achieve second-order accuracy it was necessary to develop piecewise-continuous integrators by locating the exact time of hopping to reasonably high precision. The methods differ in the way in which the spin is propagated; this can be based on NACs (rev-pc-NACs) or overlaps (rev-pc-ATDC and rev-pc-LD). These exhibit a smaller global error of the numerical integrator, , compared to the previous . This leads to a marked improvement in accuracy when calculating correlation functions and observables with large time-steps.

Additionally, there may be particular advantages in using time-reversible integrators when employing rare-event theories, such as transition-path sampling.^33^ Time-reversal symmetry can also simplify the procedure of obtaining reaction rates.^15^

These enhancements demonstrate a distinct advantage of MASH over methods like fewest-switches surface hopping (FSSH), which inherently cannot employ time-reversible integrators due to their stochastic nature. This suggests that MASH can employ a larger time-step than FSSH, making the method a more robust and efficient method for nonadiabatic molecular dynamics, in addition to the higher intrinsic accuracy exhibited in previous studies.

## Footnotes

a This is especially true when compared with commonly used approximations^16^ in FSSH which employ a size-inconsistent isotropic momentum rescaling instead of rescaling along the direction of the NAC.

b In an ab initio simulation, where there are formally an infinite number of states truncated to a set of two, it is necessary to apply Löwdin orthogonalization in order to achieve the required form.
